# Developing a framework for regular and sustainable qualitative assessment of antibiotic use in Korean medical institutions: a Delphi study

**DOI:** 10.1186/s13756-023-01319-8

**Published:** 2023-10-18

**Authors:** Se Yoon Park, Yong Chan Kim, Song Mi Moon, Bongyoung Kim, Raeseok Lee, Hong Bin Kim, Korea Study Group for Antimicrobial Stewardship (KOSGAP)

**Affiliations:** 1 Department of Internal Medicine, Hanyang University College of Medicine, 222-1, Wangsimni-ro, Seondong-gu, Seoul, 04763 Republic of Korea; 2 Division of Infectious Diseases, Department of Internal Medicine, Yongin Severance Hospital, Yonsei University College of Medicine, Yongin, Republic of Korea; 3 Department of Internal Medicine, Seoul National University Bundang Hospital, Seoul National University College of Medicine, Seongnam, 13620 Republic of Korea; 4 Department of Internal Medicine, Seoul St. Mary’s Hospital, College of Medicine, The Catholic University of Korea, Seoul, Republic of Korea

**Keywords:** Antibiotics, Qualitative assessment, Antibiotic stewardship, Consensus, Expert

## Abstract

**Background:**

We aimed to develop a roadmap for conducting regular, sustainable, and strategic qualitative assessments of antibiotic use in medical institutions within the Republic of Korea.

**Methods:**

A literature review on the current state of qualitative antibiotic assessments was conducted, followed by one open round to collect ideas, two scoring rounds to establish consensus, and one panel meeting between them. The expert panel comprised 20 experts in infectious disease or antibiotic stewardship.

**Results:**

The response rate for all three surveys was 95% (19/20), while the panel meeting attendance rate was 90% (18/20). The following long-term goals were defined to assess the annual use of antibacterial and antifungal agents in all medical institutions, including clinics. The panel agreed that random sampling of antibiotic prescriptions was the most suitable method of selecting antibiotics for qualitative assessment, with the additional possibility of evaluating specific antibiotics or infectious diseases that warrant closer evaluation for promoting appropriate antibiotic use. The plan for utilization of results from evaluation involves providing feedback while maintaining anonymity and disclosure. It includes a quantitative assessment of antibiotic prescriptions and resistance rates to compare against institutional benchmarks. Furthermore, it was agreed to link the evaluation findings to the national antibiotic stewardship programme, enabling policy and institutional approaches to address frequently misused items, identified during the evaluation.

**Conclusion:**

This study provides a framework for establishing a qualitative assessment of antimicrobial use for medical institutions at a national level in the Republic of Korea.

**Supplementary Information:**

The online version contains supplementary material available at 10.1186/s13756-023-01319-8.

## Background

Qualitative assessments of antibiotic use play an essential role in antibiotic stewardship programs (ASPs), as they not only identify patterns of inappropriate antibiotic prescribing and establish intervention strategies but also evaluate the effectiveness of these interventions [1–4]. Given that conventional qualitative antibiotic assessments require significant human resources, it is crucial to develop strategies for effectiveness based on the specific resources available in each medical environment [5].

Since the 2000s, various countries, including Australia, the United States, Europe, and the Republic of Korea (ROK), have conducted qualitative antibiotic assessments, with approximately 20–55% of antibiotic prescriptions in these countries deemed inappropriate [6–11]. Australia has been operating the National Antimicrobial Prescribing Survey since 2010 to evaluate antibiotic adequacy and has expanded the scope of diseases and medical institutions subject to qualitative antibiotic assessment [6]. In the United States, the Center for Disease Control and Prevention (CDC) has been leading the surveillance of healthcare-associated infections and qualitative antibiotic assessment since 2009 [12]. In Europe, the European Surveillance of Antimicrobial Consumption Network has conducted similar studies on both quantitative and qualitative antibiotic assessments [7, 8]. In the ROK, a nationwide qualitative antibiotic assessment began in 2018 as a project of the Korea Disease Control and Prevention Agency (KDCA), and the scope of target diseases and institutions has been expanded [2, 9, 10]. However, there are currently no standardised methods for qualitative assessment in terms of target diseases, the scope of medical institutions, and evaluation methods [2, 13]. Furthermore, despite the Korean action plan on antimicrobial resistance, ASPs in most hospitals are not well established and are mostly limited to restrictive measures for designated antimicrobials [14].

To ensure systematic and sustainable qualitative antibiotic assessments for effective implementation in ASPs, it is essential to develop a framework outlining the current assessment method and scope, as well as long-term goals for the future. Therefore, this study aimed to create a comprehensive framework for conducting regular and strategic qualitative antibiotic assessments in Korean medical institutions.

## Methods

### Overview and assembly of panel

A modified Delphi study was conducted between June and August of 2022, which included three rounds of online surveys and a virtual meeting with an expert panel. Panellists were selected to include a range of experts and policymakers involved in antibiotic use and stewardship [15]. The expert panel comprised of 20 members, including five experts from the antibiotic resistance committee in the Korean Society of Infectious Diseases, four experts from the Korea National Antimicrobial Use Analysis System (KONAS), seven infectious disease specialists with experience in ASP-related research, three experts on policy regarding antibiotic resistance representing the government, and one pharmacist from the Korea Society of Health-System Pharmacists.

### Development of survey items and the Delphi process

**Fig. 1:**
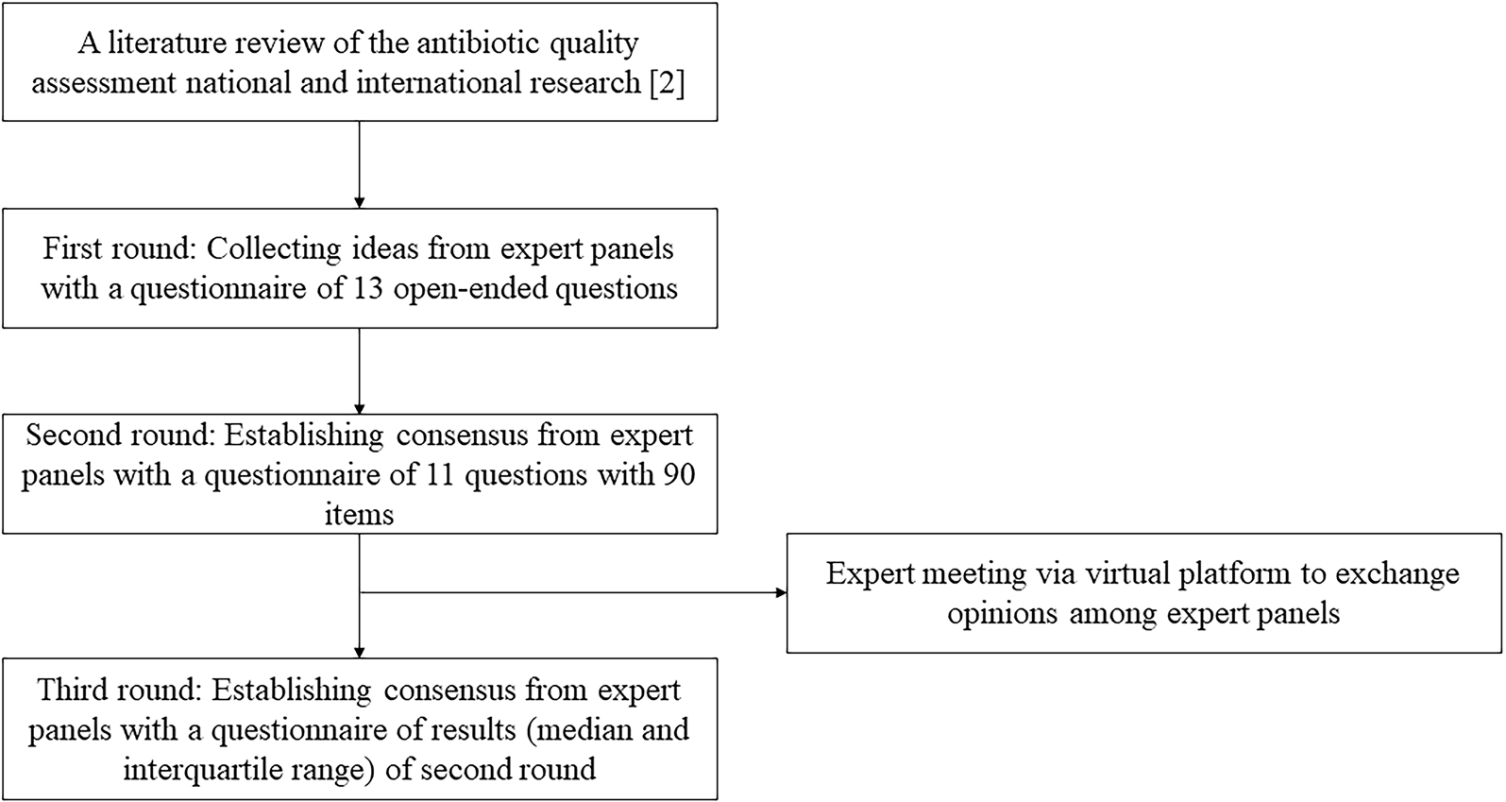
Flow diagram showing the study process

The study process is outlined in Fig. 1. An email questionnaire was sent to the expert panel members for each of the three rounds of the study, and responses were collected over a 10-days period. A reminder was sent on the 5th and 8th day of each survey to encourage participation. The survey questions were formulated following discussion among study team members (SYP, YCK, SMM, BK, RL, and HBK), drawing from a literature review of qualitative antibiotic assessment systems and researches from Europe, the US, Australia, ROK, and the global point prevalence survey of antimicrobial consumption and resistance (Global-PPS) [2]. The literature review results were summarised in the first survey to provide basic information for the subsequent surveys.

The first round of the survey aimed to gather insights and ideas from the expert panel. A questionnaire consisting of open-ended questions and a summary of the literature review were distributed to the panel members via email (Additional file 1). The questionnaire for the second survey was developed based on the panel members’ responses to the first survey. The experts’ responses for each item were evaluated using a 5-point Likert scale. The scale ranged from 1, indicating strong disagreement, to 5, indicating strong agreement, with 4 and 5 considered to signify ‘agree’. The panel members were also invited to add their opinions on each question. A virtual meeting was held between the second and third surveys, and all panel members were invited to participate. The meeting's agenda focused on exchanging opinions regarding the questionnaire items present in the second survey. A report with the results of the second survey was sent to all participants before the meeting. The results of the second survey were displayed in the third questionnaire, with central tendency and dispersion shown as median and interquartile ranges. In the third round, the panel members’ opinions on each item were again evaluated using a 5-point Likert scale. Respondents were invited to comment if their opinions fell outside the majority of the other experts' estimates.

### Validation of items

We calculated content validity ratios (CVRs) to analyse the responses provided in the second and third surveys and select the items that showed the highest levels of agreement among the panel members. The formula used to calculate CVR was as follows: CVR = (n_e_ − N/2)/(N/2), where ‘n_e_’ represents the number of panel experts who rated a given item as ‘agree’, and ‘N’ represents the total number of panellists. CVR values ranged from − 1 to + 1, with higher values indicating greater agreement among experts. CVR values above the cut-off level (minimum CVR values) were generally considered to have achieved a sufficient level of agreement. The minimum CVR values were determined based on the number of experts participating in each round, which were 0.44 for 18 participants and 0.47 for 19 participants [16, 17]. We used minimum CVRs as consensus criteria. The consensus was achieved after fixed three-round survey.

## Results

**Fig. 2:**
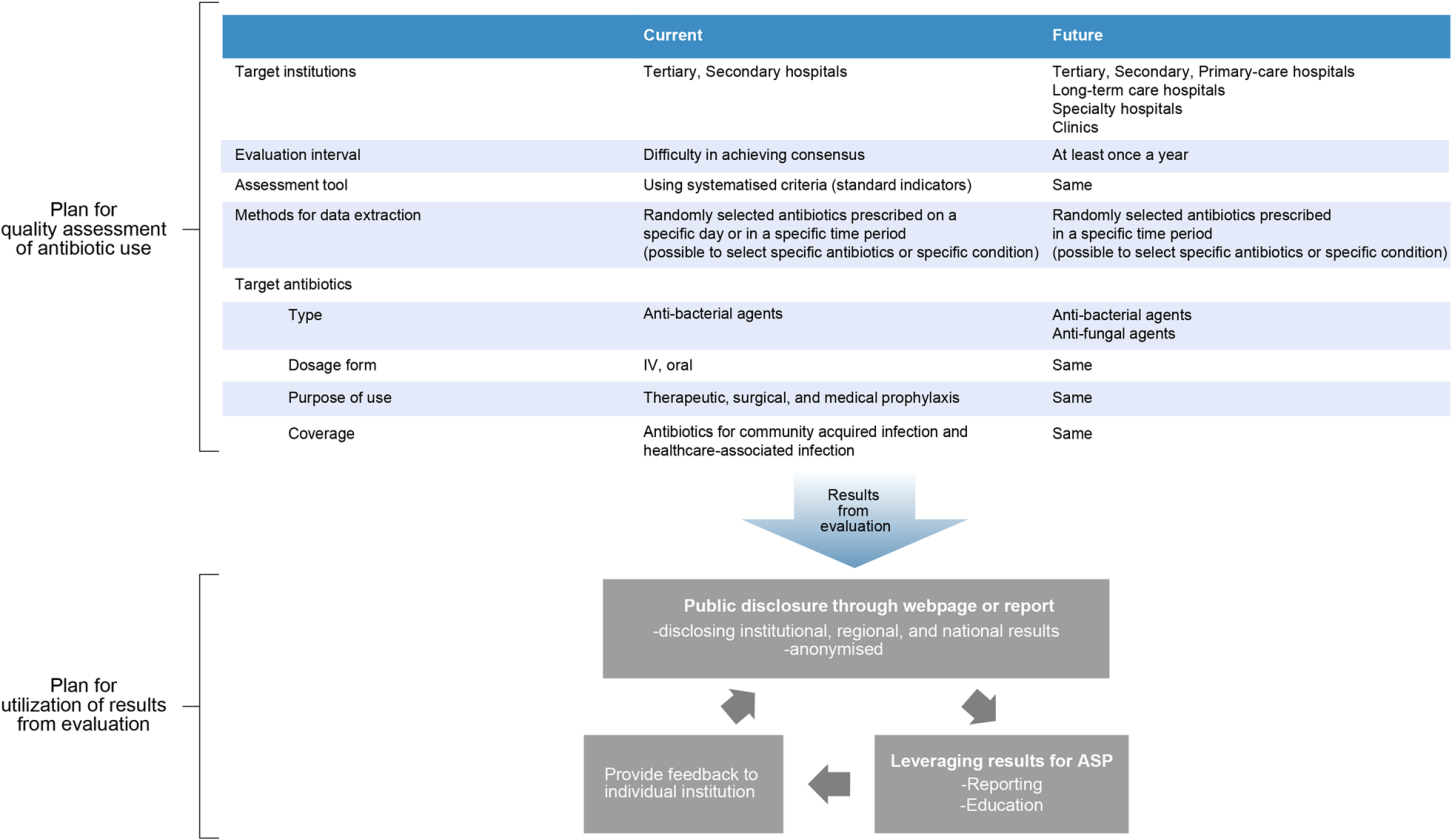
Roadmap for assessing antibiotic utilisation and leveraging evaluation outcomes

The response rates for the first, second, and third rounds were all 95% (19/20). Eighteen expert panellists (90%) participated in the virtual meeting (Additional file 2: Table S1). One panellist did not complete the survey and provided partial answers; as a result, we applied different CVR standards for each item. The summarised results are presented in Fig. 2 and Additional file 2: Tables S2 and S3.

### Practical challenges in conducting qualitative antibiotic assessments

The most frequently reported practical challenges in conducting qualitative antibiotic assessments included inadequate financial compensation (mean score 4.95), a shortage of qualified personnel, lack of institutional support (both scoring 4.84), limited awareness among management (4.79), the absence of computerised programs (4.42), inadequate long-term planning (4.05), and lack of agreed-upon evaluation criteria (3.84).

### Current options and long-term goals for antibiotic quality assessments

Table 1 displays the Delphi survey results regarding antibiotic quality assessment operators, cycles, and target organisations. All experts agreed that the KDCA or its professional governmental organisations should manage antibiotic quality assessments. Most experts agreed that the current appropriate interval for antibiotic quality assessments is at least once a year (13/19); however, the agreement rate did not reach the consensus standard (CVR = 0.368). Conversely, out of 19 experts, 16 agreed that antibiotic quality assessments should be conducted at least once a year in the future, reaching a consensus (CVR = 0.684). As for the scope of target institutions, the experts agreed that tertiary care hospitals (19/19, CVR = 1.000), secondary care hospitals (18/19, CVR = 0.895), and hospitals with 500 or more beds (19/19, CVR = 1.000) are currently the most appropriate, whereas specialty hospitals (19/19, CVR = 1.000), clinics (16/19, CVR = 0.684), and hospitals with 300 or more beds (18/19, CVR = 0.895) should be included in the future.

**Table 1:** Results of the Delphi survey on operator, cycle, and target organisations for qualitative antibiotic assessments

Items	Agree (%)	Disagree (%)	Neutral (%)	CVR
*Operator for the antibiotic quality assessment*				
Individual medical institutions	1 (5.3)	7 (36.8)	11 (57.9)	− 0.895
Professional societies (KSID, KSAC, etc.)	11 (57.9)	2 (10.5)	6 (31.6)	0.158
The Center for Disease Control and Prevention or its professional organisations	19 (100.0)	0	0	1.000
Korea Health Insurance Organization	1 (5.3)	12 (63.2)	6 (31.6)	0.158
*Appropriate intervals for performing routine antibiotic quality assessments (currently)*				
At least twice a year	0	18 (94.7)	1 (5.3)	− 1.000
At least once a year	13 (68.4)	3 (15.8)	3 (15.8)	0.368
Every 2 years	5 (26.3)	4 (21.1)	10 (52.6)	− 0.474
Every 3–5 years	5 (26.3)	12 (63.2)	2 (10.5)	− 0.474
Occasionally	1 (5.3)	15 (78.9)	3 (15.8)	− 0.895
*Appropriate intervals for performing routine antibiotic quality assessments (in the future)*				
At least twice a year	3 (15.8)	8 (42.1)	8 (42.1)	− 0.684
At least once a year	16 (84.2)	1 (5.3)	2 (10.5)	0.684
Every 2 years	8 (42.1)	4 (21.1)	7 (36.8)	− 0.158
Every 3–5 years	1 (5.3)	13 (68.4)	5 (26.3)	− 0.895
Occasionally	1 (5.3)	17 (89.5)	1 (5.3)	− 0.895
*Scope of target institutions to be included by the type of hospital (currently)*				
Tertiary care hospitals	19 (100.0)	0	0	1.000
Secondary care hospitals	18 (94.7)	0	1 (5.3)	0.895
Primary care hospitals	5 (26.3)	3 (15.8)	11 (57.9)	− 0.474
Long-term care hospitals	1 (5.3)	12 (63.2)	6 (31.6)	− 0.895
Specialty hospitals (orthopaedics, ophthalmology, etc.)	0	5 (27.8)	13 (72.2)	− 1.000
Clinics	0	18 (94.7)	1 (5.3)	− 1.000
*Scope of target institutions to be included by the number of hospital beds (currently)*				
Hospitals with 1000 + beds	19 (100.0)	0	0	1.000
500 + beds	19 (100.0)	0	0	1.000
300 + beds	3 (15.8)	2 (10.5)	14 (73.7)	− 0.684
*Scope of target institutions to be included by the type of hospital (in the future)*				
Tertiary care hospitals	19 (100.0)	0	0	1.000
Secondary care hospitals	19 (100.0)	0	0	1.000
Primary care hospitals	19 (100.0)	0	0	1.000
Long-term care hospitals	19 (100.0)	0	0	1.000
Specialty hospitals (orthopaedics, ophthalmology, etc.)	19 (100.0)	0	0	1.000
Clinics	16 (84.2)	1 (5.3)	2 (10.5)	0.684
*Scope of target institutions to be included by the number of hospital beds (in the future)*				
Hospitals with 1000 + beds	19 (100.0)	0	0	1.000
500 + beds	19 (100.0)	0	0	1.000
300 + beds	18 (94.7)	0	1 (5.3)	0.895

*CVR* content validity ratio, *KSID* Korean Society of Infectious Diseases, *KSAC* Korean Society of Antimicrobial Therapy

Table 2 presents the Delphi survey results concerning the types of antibiotics to include in the quality assessments. Experts reached a consensus on systemic antibiotics (19/19, CVR = 1.000) and antibacterial agents (19/19, CVR = 1.000) as the appropriate range for the current quality assessments and agreed that antifungal agents (16/18, CVR = 0.778) should be included in the future. By contrast, antiviral, antitubercular, antiprotozoal, antimalarial, and locally administered antibiotics were not considered within the scope of the survey. Experts agreed that all types of antibiotics should be included in the assessment, regardless of prescription purpose. Regarding the method of extracting antibiotic use information for evaluation, the highest agreement rate was for the assessment of randomised samples rather than a full survey during a given period (19/19, CVR = 1.000). Combining an assessment of antibiotics prescribed within a specific period with an assessment of specific antibiotics or conditions as needed (18/19, CVR = 0.895) were identified as short-term and long-term plans, respectively.

**Table 2:** Results of the Delphi survey on antimicrobial agents to be included and on methods for data extraction for antibiotic quality assessment

Items	Agree (%)	Disagree (%)	Neutral (%)	CVR
*The range of antimicrobial agents that can be included (currently)*				
Antibacterial agents	19 (100.0)	0	0	1.000
Antifungal agents	6 (33.3)	1 (5.6)	11 (61.1)	− 0.333
Antiviral agents	2 (11.1)	5 (27.8)	11 (61.1)	− 0.778
Anti-TB, protozoal, malarial agents	0	12 (66.7)	6 (33.3)	− 1.000
Systemic antibiotics (IV, oral)	19 (100.0)	0	0	1.000
Local antibiotics (nebuliser, ointment, etc.)	1 (5.3)	12 (63.2)	6 (31.6)	− 0.895
*The range of antimicrobial agents that can be included (in the future)*				
Antibacterial agents	19 (100.0)	0	0	1.000
Antifungal agents	16 (88.9)	0	2 (11.1)	0.778
Antiviral agents	12 (66.7)	2 (11.1)	4 (22.2)	0.333
Anti-TB, protozoal, malarial agents	2 (11.1)	4 (22.2)	12 (66.7)	− 0.778
Systemic antibiotics (IV, oral)	19 (100.0)	0	0	1.000
Local antibiotics (nebuliser, ointment, etc.)	3 15.8	9 47.4	7 36.8	− 0.684
*Types of antibiotics based on the purpose of the antibiotic prescription (currently)*				
Therapeutic	19 (100.0)	0	0	1.000
Surgical prophylaxis	19 (100.0)	0	0	1.000
Medical prophylaxis	17 (89.5)	0	2 (10.5)	0.789
Antibiotics for community acquired infection	19 (100.0)	0	0	1.000
Antibiotics for healthcare-associated infection	19 (100.0)	0	0	1.000
*Types of antibiotics based on the purpose of the antibiotic prescription (in the future)*				
Therapeutic	19 (100.0)	0	0	1.000
Surgical prophylaxis	19 (100.0)	0	0	1.000
Medical prophylaxis	19 (100.0)	0	0	1.000
Antibiotics for community acquired infection	19 (100.0)	0	0	1.000
Antibiotics for healthcare-associated infection	19 (100.0)	0	0	1.000
*Methods for quality assessment of antibiotics (based on expert opinion, specific criteria [quality indicators])*				
Evaluation based on expert judgement (not based on specific criteria)	10 (52.6)	2 (10.5)	7 (36.8)	0.053
Evaluation using systematised criteria (quality indicators)	19 (100.0)	0	0	1.000
The need for regular revision of quality indicators developed through consensus of domestic expert groups	19 (100.0)	0	0	1.000
*How to extract targeted antibiotics for inclusion in a qualitative antibiotic assessment (currently)*				
Evaluation of a random sample of antibiotics prescribed on a given day (without considering the duration of antibiotic use)	15 (78.9)	2 (10.5)	2 (10.5)	0.579
Evaluation of all antibiotics prescribed on a given day (without considering the duration of antibiotic use)	4 (21.1)	5 (26.3)	10 (52.6)	− 0.579
Randomised evaluation of antibiotics prescribed in a specific period (considering the duration of antibiotic use)	19 (100.0)	0	0	1.000
Evaluation of all antibiotics prescribed in a specific period (considering the duration of antibiotic use)	6 (31.6)	3 (15.8)	10 (52.6)	− 0.368
Evaluation of antibiotics prescribed at a specific point in time for a specific disease or for a specific antibiotic (census or randomised)	14 (77.8)	2 (11.1)	3 (16.7)	0.556
Combining an assessment of antibiotics prescribed in a specific period with an assessment of specific antibiotics or conditions as needed (all or randomised)	16 (84.2)	2 (10.5)	1 (5.3)	0.684
*How to extract targeted antibiotics for inclusion in a qualitative antibiotic assessment (in the future)*				
Evaluation of a random sample of antibiotics prescribed on a given day (without considering the duration of antibiotic use)	8 (42.1)	3 (15.8)	8 (42.1)	− 0.158
Evaluation of all antibiotics prescribed on a given day (without considering the duration of antibiotic use)	9 (47.4)	4 (21.1)	6 (31.6)	− 0.053
Randomised evaluation of antibiotics prescribed in a specific period (considering the duration of antibiotic use)	18 (94.7)	0	1 (5.3)	0.895
Evaluation of all antibiotics prescribed in a specific period (considering the duration of antibiotic use)	17 (89.5)	1 (5.3)	1 (5.3)	0.789
Evaluation of antibiotics prescribed at a specific point in time for a specific disease or for a specific antibiotic (census or randomised)	8 (42.1)	1 (5.3)	10 (52.6)	− 0.158
Combining an assessment of antibiotics prescribed in a specific period with an assessment of specific antibiotics or conditions as needed (all or randomised)	18 (94.7)	1 (5.3)	0	0.895

*CVR* content validity ratio, *IV* intravenous, *TB* tuberculosis

### Reporting/feedback on antibiotic quality assessment results

Regarding ways to encourage organisations to participate in antibiotic quality assessments, ‘establishing payment/incentives (19/19, CVR = 1.000) and incorporating the assessment into medical quality assessment or accreditation evaluation (19/19, CVR = 1.000) were strongly recommended by the panellists. For reporting/feedback methods, there was a strong consensus for anonymised disclosure through reports with quantitative assessments of antibiotic use and resistance rates and for individual healthcare organisations to view their results through a private website (19/19, CVR = 1.000). By contrast, publishing quality assessment results to the public or disclosing regional and national results to sites that reveal individual evaluation results of medical institutions, such as Health Insurance Review and Assessment Service (HIRA), was not preferred (0/19, CVR = − 1.000 and 5/19, CVR = − 0.474, respectively).

### Strategies for linkage between the antibiotic assessment results and antimicrobial stewardship programs

Regarding strategies for linkage to ASPs, there was a positive consensus on reporting quality assessment results to management and staff within healthcare organisations (19/19%, CVR = 1.000). Additionally, a policy and regulatory approach (such as providing incentives, including in healthcare quality evaluations) and the implementation of healthcare professional education initiatives (such as mandatory education for medical associations) for frequent misuse of antibiotics identified in qualitative evaluation results were agreed upon by the experts (19/19, CVR = 1.000). While there was positive consensus regarding public disclosure and the promotion of qualitative analysis results on antibiotic use among the general population, three experts remained neutral (16/19, CVR = 0.684; Table 3).

**Table 3:** Results of the Delphi survey on a plan to involve medical institutions in qualitative antibiotic assessments and a plan to utilise these results

Items	Agree (%)	Disagree (%)	Neutral (%)	CVR
*Engaging organizations in qualitative antibiotic assessment*				
Reflect ‘antibiotic quality assessment’ in medical quality assessment	19 (100.0)	0	0	1.000
Reflect ‘antibiotic quality assessment’ in medical centre accreditation evaluation	19 (100.0)	0	0	1.000
Antibiotic stewardship fee/incentive payment	19 (100.0)	0	0	1.000
Establish mandatory provisions for hiring personnel in charge of antibiotic quality assessment	17 (89.5)	0	2 (10.5)	0.789
Provide penalties for non-participating organizations (compulsory participation)	0	18 (94.7)	1 (5.3)	− 1.000
Minimise labour input by individual institutions (e.g., evaluation using data from the Korea Health Insurance Corporation or the Korea Health Insurance Review and Assessment Service)	9 (47.4)	4 (21.1)	6 (31.6)	− 0.053
*Reporting/feedback methods for antibiotic quality assessment results*				
Publishing individual institutions’ inappropriate prescribing rates in an annual report or on a public website that is open to the public	0	16 (84.2)	3 (15.8)	− 1.000
Maintaining a website where individual healthcare organisations can view only their institution’s results	13 (68.4)	0	6 (31.6)	0.368
Allowing individual providers to view institutional, regional, and national results through a private website (anonymised without disclosing provider names)	19 (100.0)	0	0	1.000
Disclosure of qualitative evaluation results along with quantitative evaluation and antibiotic resistance rates through the Antibiotic Use/Resistance Report, which is open to the public (anonymised without disclosing the name of the institution)	19 (100.0)	0	0	1.000
Disclose regional and national results by adding them as indicators to sites that disclose individual evaluation results of medical institutions, such as the Health Insurance Review and Assessment Service website (individual institution results are not disclosed)	5 (26.3)	5 (26.3)	9 (47.4)	− 0.474
*How antibiotic quality assessments can be linked to an antibiotic stewardship program*				
Individual healthcare organisation representatives reporting quality assessment results to leadership/staff within the healthcare organisation	19 (100.0)	0	0	1.000
Publicising and disseminating the results of the antibiotic quality assessments to the general public	16 (84.2)	0	3	0.684
Implement healthcare provider education on common antibiotic misuse identified in the quality assessment (e.g., mandatory medical association education)	19 (100.0)	0	0	1.000
Policy and regulatory approaches (e.g., incentives, inclusion in healthcare quality measures, etc.) to address common antibiotic misuse identified in the quality assessment results	19 (100.0)	0	0	1.000

*CVR* content validity ratio

## Discussion

The significance of this study lies in the establishment of goals and detailed methods for conducting qualitative assessment of antibiotics, both today and long-term. Given the absence of standard recommendations for qualitative antibiotic assessments, the experts who participated in the survey were presented with various approaches currently being used in different countries. By identifying the strengths and weaknesses of these approaches, a more objective application of qualitative antibiotic assessments in the ROK can hopefully be achieved.

The consensus reached regarding the long-term goal for qualitative assessment of antibiotics involved conducting annual assessments by the KDCA for antibacterial and antifungal agents. Antibiotics prescribed for specific periods will be assessed through random sampling, and evaluations will be conducted for particular antibiotics or diseases as needed. The short-term goals largely align with the long-term goals, except that primary hospitals, clinics, specialised medical institutions, and hospitals with fewer than 300 beds will not be included in qualitative antibiotic assessments. These findings indicate that infectious disease or ASP experts in the ROK acknowledge the significance of qualitative assessments of antibiotics, even at present, and agree on the necessity of expanding the assessments to eventually include clinics. There is currently no consensus regarding the frequency of antibiotic evaluations, with experts agreeing on an annual evaluation for the long term. In the short term, annual evaluations are deemed unfeasible owing to resource constraints. It may be necessary to initiate a 2–5 years cycle, but the long-term goal is to gradually increase the frequency of evaluations to an annual basis, as is currently done in Australia [6].

Determining how to select the antibiotics to be evaluated is crucial, as it is connected to the required resources. In this study, there was a long-term consensus on using random sampling while also incorporating time periods. Evaluating all patients would be labour-intensive; therefore, random sampling presents a sustainable assessment method in the long run. In the ROK, qualitative assessments of antibiotic prescriptions were conducted in 2018 and 2019 using a 1-day complete enumeration and a 2-days random sampling method, respectively. Despite including more institutions in the second survey, the rates of inappropriate antibiotic prescriptions were similar in both surveys [2, 9].

Evaluating the duration of antibiotic use is an important aspect of qualitative assessment but is difficult to conduct using point surveillance methods [2]. In 2021 and 2022, disease-specific evaluations for urinary tract infections and bacteraemia were conducted, and the appropriate antibiotic prescription period was determined by evaluating the entire period. This approach overcame the challenge of assessing many quality indicators with other point surveillance methods [2]. It was agreed that specific quality indicators should be used for assessment, which cannot rely solely on expert judgement, and continuous updates are necessary [18].

The lack of expert consensus on using HIRA data for qualitative assessment of antibiotic prescriptions to minimise the labour input required from individual institutions is noteworthy, as only 47.4% of respondents agreed to its use. This could be due to concerns surrounding code shifting and the unavailability of clinical data in the HIRA database, as previously reported [19]. The survey results emphasised the need for significant human resources for qualitative assessments of antibiotics and the importance of actively seeking funding to recruit personnel for this purpose. However, since manpower alone would not cover all institutions, evaluation based on a national database method should be considered as a supplementary measure for some areas [20–22].

It was agreed that after assessments are conducted, the feedback method should involve anonymising and publicly disclosing the data, as well as including it in the antibiotic use/resistance report. In the ROK, KONAS conducts quantitative assessments of antibiotics, and annual antibiotic resistance reports are published through Kor-GLASS, the Korean national antimicrobial resistance surveillance system based on the GLASS platform [23–25]. The integration of reported data is expected to identify the impact of ASP activity on the antibiotic resistance rate through both quantitative and qualitative antibiotic assessments. If the same analysis is also performed on healthcare-associated infection rates, as is done in the US, it may serve as an indicator of the effect of the ASP [12].

It is noteworthy that the entire expert panel agreed on the utilization of the results of the qualitative assessment for the national ASP. The qualitative assessment results enable physicians to identify antibiotics that are being prescribed incorrectly and can be used as evidence to improve the use of those antibiotics. The qualitative assessment implemented at a national level provide an opportunity to reduce the amount of antibiotics used inappropriately, which may be related to decrease in antibiotic resistance. It is expected that the integration of antibiotic qualitative assessment into healthcare quality assessment or the introduction of incentive systems will increase the interest and participation of medical institutions in ASPs. However, three experts in this Delphi survey took a neutral attitude to public disclosure and publicizing the results of the qualitative assessment. Therefore, the strategy for linking the results of the qualitative assessment and the national ASP needs to be discussed further.

This study had some limitations. Firstly, there are several unanswered questions about conducting a large-scale, population-based assessment of antibiotic appropriateness. We did not discuss what criteria should be used to assess antibiotic appropriateness, how many cases should be sampled from each hospital, or who should actually perform the antibiotic appropriateness audits. Further research is warranted to address these questions. Secondly, the international applicability of this study is limited because the survey was only administered to experts in the ROK. However, the study framework itself could be used as a template in other countries, and the methodology presented could also be used to make country-specific adjustments. Lastly, consensus/closing criteria applied in studies using Delphi procedure are varied because there is no standard for that criteria [26]. In this study, we used CVRs, which are widely used to quantify content validity, and tried to effectively reflect the opinions of experts with consensus criteria, considering the number of experts.

## Conclusions

This Delphi survey established an expert consensus regarding both short- and long-term roadmaps for conducting qualitative assessments of antibiotics in the ROK. The plan for quality assessment of antibiotic use included target institutions, evaluation interval, assessment tool, methods for data extraction, and target antibiotics. The plan for utilization of results from evaluation included engagement in qualitative antibiotic assessment, reporting/feedback methods, and ASP linkage. This practice has not yet been standardised worldwide. Its outcomes are expected to promote active qualitative assessments of antibiotics and to appropriately link evaluation results to individual medical institutions and the national ASP.

### Supplementary Information

**Additional file 1:** A questionnaire consisting of open-ended questions and a summary of the literature review.

**Additional file 2**. **Table S1**: The response rate of the expertise panel in each round. **Table S2**: Proposed plan for qualitative assessment of antibiotic use. **Table S3**: How to best use assessment data.

## Data Availability

The datasets analysed during the current study are available from the corresponding author on reasonable request.

## Abbreviations

ASP: Antibiotic stewardship programs
HIRA: Health Insurance Review and Assessment
IRB: Institutional review board
KDCA: Korea Disease Control and Prevention Agency
KONAS: Korea National Antimicrobial Use Analysis System
ROK: Republic of Korea
