# Supplementary material for: Developing a framework for regular and sustainable qualitative assessment of antibiotic use in Korean medical institutions: a Delphi study
Source: Antimicrob Resist Infect Control. 2023 Oct 18;12:114. doi: 10.1186/s13756-023-01319-8 (PMC10585816; doi:10.1186/s13756-023-01319-8)
Supplement: Supplementary file 1 — Additional file 1: A questionnaire consisting of open-ended questions and a summary of the literature review. [file 13756_2023_1319_MOESM1_ESM.docx]

**The first Delphi study survey on the development of a framework for the qualitative assessment of antibiotic use in the Republic of Korea (ROK)**

Following the pilot project in 2018 as a policy research service project of the Korea Disease Control and Prevention Agency (KDCA), the ‘research on the development of a framework for the qualitative assessment of antibiotic prescriptions in Korean medical institutions and antibiotic quality assessment at the national level’ has been implemented since 2019. This research team aims to develop a plan (framework) for the qualitative assessment of antibiotic use suitable for the Korean situation to continuously identify and improve targets for improving antibiotic prescriptions. We plan to conduct two Delphi surveys and face-to-face meetings. We would appreciate your active participation and opinions.

This survey will be limited to the ‘qualitative assessment of antibiotic use in humans’ and will not cover ‘antibiotic use in animals’ or ‘quantitative assessment of antibiotic use’.

References

<Major results related to the study on the qualitative assessment of antibiotic use in the ROK>

- 2018: When a qualitative assessment was conducted on all antibiotics prescribed in a day at 20 medical institutions, the rate of inappropriate antibiotic prescriptions was 27.7%.

- 2019: A qualitative assessment was conducted by extracting some samples twice from the antibiotics prescribed in a day at 75 Korean medical institutions. The rate of inappropriate antibiotic prescriptions was 26.1%.

- 2021: A qualitative assessment was conducted on antibiotics prescribed for asymptomatic bacteriuria and urinary tract infections in 26 hospitals in ROK. The rate of inappropriate antibiotic prescriptions was approximately 20–30%.

- 2022: A qualitative assessment is being implemented on antibiotic use in patients with bacteraemia.

<Current status of qualitative assessment of antibiotics by country>

| United States | - In 2011 and 2015, the CDC led a surveillance study of the current status of healthcare-associated infectious diseases and antibiotic use status. Between 2015 and 2016, a qualitative assessment of antibiotic prescription was conducted in 199 hospitals in 10 states. |
| --- | --- |
| Australia | - As part of the National Antimicrobial Prescribing Survey (NAPS) organised by the Melbourne Doherty Institute, qualitative assessments of antibiotic use in hospitals began in 2010 and long-term care hospitals in 2015. In addition, a qualitative assessment of antibiotic use for surgical site infections began in 2016.  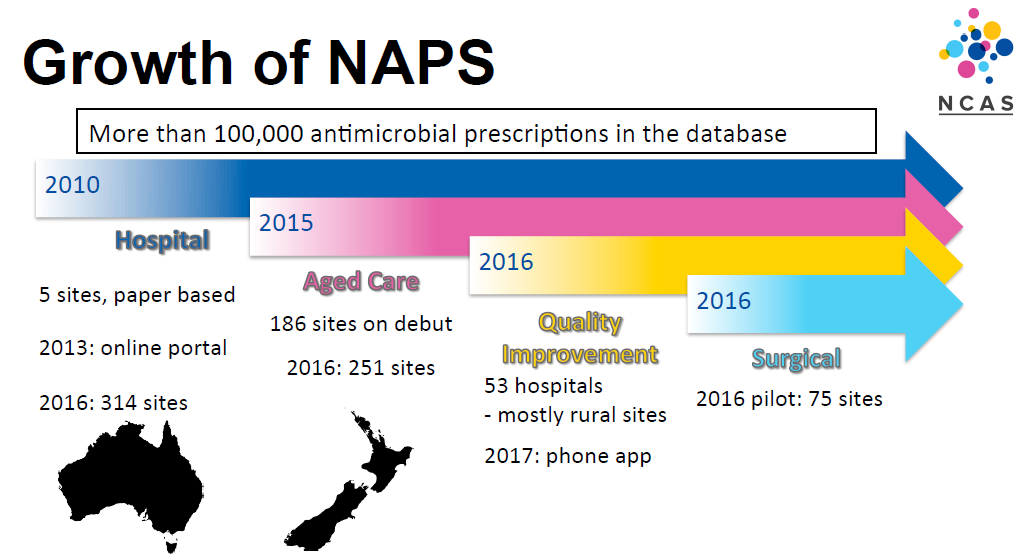 |
| Europe | - No qualitative assessment for antibiotic use has been conducted at the national level. In some individual studies, the current status of antibiotic use has been identified and qualitative assessments have been conducted. |
| Global PPS project | - A qualitative assessment was conducted on hospitals worldwide willing to participate. Antibiotic selection in compliance with the guidelines was evaluated, and definitive antibiotics were evaluated by inputting the antibiotic susceptibility results of the identified bacteria. |

**1. Basic information about the participant**

- Name ( )

- Institution ( )

- Position ( )

**2. We would like to ask for your opinion on the necessity of routine antibiotic quality assessments in Korean medical institutions and the practical difficulties of their realisation.**

- Reasons for antibiotic quality assessment at present (e.g., it is necessary to understand the current status of antibiotic misuse and abuse) ( )

- Practical difficulties at present (e.g., insufficient number of professionals to perform the assessment)

( )

**3. We would like to ask for your opinion on the most appropriate interval for routine antibiotic quality assessments if these are performed.**

| Examples |
| --- |
| - Regular assessment every year (ROK, Australia)  - Assessment of major antibiotics and syndromes at intervals of several years (4 years) (United States) |

- Interval that can be implemented at present ( )

- Long-term/ideal goals ( )

- We would like to ask you for any other opinions related to this topic. ( )

**4. We would like to ask for your opinions on the operating body for the routine antibiotic quality assessments (finance and operation).**

| Examples |
| --- |
| - Led by individual medical institutions  - Professional societies associated with infections or antibacterial agents (the Korean Society of Infectious Diseases [KSID]/ Korean Society for Antimicrobial Therapy [KSAT], etc.)  - Korea Disease Control and Prevention Agency (KDCA)  - National Health Insurance Service (NHIS) |

| ROK (policy research service project) | - Policy research service project of the Korea Centers for Disease Control and Prevention (KCDC) (2019-2022) |
| --- | --- |
| United States | - Led by the CDC, it is implemented utilising the Emerging Infectious Programs (EIP) in 10 states in the United States. |
| Australia | - The National Antimicrobial Prescribing Survey (NAPS) is operated by the National Centre for Antimicrobial Stewardship (NCAS), a multidisciplinary team affiliated with the Melbourne Doherty Institute |
| Europe | - Although no assessment has been implemented at the national level, there have been projects to develop standard indicators for adequacy assessment.  - European Surveillance of Antimicrobial Consumption-Network (ESCA-Net)  - Driving Reinvestment in Research and Development and Responsible Antibiotic Use (DRIVE-AB) |
| Global PPS project | - Herman Goossens (University Hospital of Antwerp, Belgium) |

- Possible operator at present ( )

- Long-term/ideal goals ( )

- We would like to ask you for any other opinions related to this topic. ( )

**5. We would like to ask for your opinions on the appropriate scope of institutions for antibiotic quality assessment.**

| Examples |
| --- |
| - Tertiary care hospitals, secondary care hospitals, primary-care hospitals, and clinics  - Classified by the number of hospital beds (less than 300 beds, 300–500 beds, 500–1,000 beds, and more than 1,000 beds) |

- Appropriate target institution at present ( )

- Long-term/ideal goals ( )

| ROK (policy research service project) | - 2018: 20 medical institutions nationwide – tertiary care hospitals (10), secondary care hospitals (9), long-term care hospitals (1)  - 2019: 75 medical institutions nationwide – tertiary care hospitals (37), secondary care hospitals (36), hospitals (1), long-term care hospitals (1)  - 2019: Targeting local small and medium-sized hospitals (6 long-term care hospitals, 3 acute hospitals, and 1 orthopaedic hospital)  - 2020: 26 medical institutions nationwide – tertiary care hospitals (13), secondary care hospitals (11), long-term care hospitals (2)  - 2022: 27 medical institutions nationwide – tertiary care hospitals (17), secondary care hospitals (10) |
| --- | --- |
| United States | - 199 medical institutions affiliated with EIP in 10 states in the United States (96 acute hospitals with <150 beds, 76 with 15–399 beds, and 27 with ≥400 beds) |
| Australia | - Hospital NAPS: as acute and subacute (private and public) hospitals are the target medical institutions, the number of participating hospitals gradually increased, with 377 hospitals (268 public and 109 private hospitals) participating in 2019.  - Long-term Care NAPS: all long-term care facilities in Australia that are willing to participate voluntarily. Since 2017, long-term care facilities in the Victorian government have been required to participate as part of the VICNISS Infection Control Indicator Programme. As of 2019, a total of 568 long-term care facilities were participating.  - Surgical NAPS: acute and subacute (private and public) hospitals performing surgeries and procedures. As of 2019, 144 hospitals (74 public and 70 private hospitals) participated. |
| Europe | [EASC PPS]  - In 2006, PPS was implemented in 20 hospitals in 20 European countries.  - In 2008, PPS was implemented in 32 hospitals in 21 European countries.  - In 2009, PPS was implemented in 172 hospitals in 25 European countries.  - In 2006 and 2009, two PPSs were implemented in 28 European countries, with a total of 186 hospitals participating. |
| Global PPS project | - All hospitals admitting inpatients worldwide and willing to participate in the global PPS are targeted. |

- We would like to ask you for any other opinions related to this topic. ( )

**6. We would like to ask for your opinion on the types of antimicrobial agents to be evaluated in the antibiotic quality assessments (e.g., antibiotics, antifungal agents, antiviral agents, antiprotozoal agents, and antimalarial agents).**

| ROK (policy research service project) | - Antibiotics and antifungal agents (antiviral agents are not included) |
| --- | --- |
| United States | - Antibiotics, antifungal agents, antiviral agents, and specific antituberculosis agents are all included in the analysis |
| Australia | - Hospital NAPS: systemic and topical antibiotics (antibacterials) and antifungal and antiviral agents are included.  - Long-term Care NAPS: systemic and topical antibiotics (antibacterials) and antifungal and antiviral agents are all included.  - Surgical NAPS: systemic and topical antibiotics (antibacterials) |
| Europe | [EASC PPS]  - 2006: systemic antimicrobial therapy according to the WHO ATC classification (J01, J04AB (rifampicin only), A07AA (oral vancomycin and colistin only), and P01AB (oral metronidazole only))  - 2008: antimicrobial agents (J01), antifungal agents (J02), rifampicin (J04AB02, excluding the indication for antituberculosis), oral vancomycin and colistin (A07AA09-10), and oral and rectal metronidazole (P01AB01) for systemic use according to the WHO ATC classification  - 2009: systemic antimicrobial agents, including antifungal agents  - 2008, 2009: antimicrobial agents of J01, J02, A07AA, P01AB, D01BA, and J04AB02 according to the WHO ATC classification (rifampicin is included for all indications except for tuberculosis) and antifungal agents (J02) are included in the survey. |
| Global PPS project | - Antibiotics, antifungal agents, antituberculosis agents, antiprotozoal agents, antiviral agents, and antimalarial agents are all included, whereas topical antibiotics are excluded. |

- Types of antimicrobial agents that can be included at present ( )

- Long-term/ideal goals ( )

- We would like to ask you for any other opinions related to this topic. ( )

**7. We would like to ask for your opinion on which antibiotic use purposes should be evaluated in the antibiotic quality assessment (e.g., therapeutic, surgical prophylaxis, and medical prophylaxis).**

| ROK (policy research service project) | - 2018, 2019: therapeutic, surgical prophylaxis, and medical prophylaxis  - 2020: asymptomatic bacteriuria/urinary tract infection  - 2022: bacteraemia |
| --- | --- |
| United States | - 2011: therapeutic antibiotics  - 2015: therapeutic, surgical prophylaxis, and medical prophylaxis |
| Australia | - Hospital NAPS: depending on the indications for antibiotic use, antibiotics are classified into therapeutic, surgical prophylactic, and medical prophylactic antibiotics.  - Long-term Care NAPS: the prevalence of infectious diseases and the use of antibiotics among patients in long-term care facilities are mainly evaluated.  - Surgical NAPS: antibiotics used prophylactically before and after surgery are being evaluated. |
| Europe | - Studies including both therapeutic and prophylactic antibiotics have been mainly performed. |
| Global PPS project | - According to the purpose of antibiotic use, antibiotics are classified as follows: antibiotics for community-acquired infection, antibiotics for healthcare-associated infection, surgical prophylactic antibiotics, medical prophylactic antibiotics, and others |

- Antibiotic types that can be included at present ( )

- Long-term/ideal goals ( )

- We would like to ask you for any other opinions related to this topic. ( )

**8. We would like to ask for your opinion on methods for selecting subjects for antibiotic quality assessments.**

| Examples |
| --- |
| - Point surveillance study (without considering the duration of antibiotic use) vs assessment considering the duration of antibiotic use  - Complete enumeration vs. random sampling |

| ROK (policy research service project) | - 2018: point surveillance study (1 day, complete enumeration)  - 2019: point surveillance study (2 days, random sampling)  - 2020: asymptomatic bacteriuria/urinary tract infection, including period assessment (1 month, complete enumeration)  - 2022: bacteraemia, including period assessment (2 months, complete enumeration) |
| --- | --- |
| United States | - 2015: point surveillance study (1 day selected between May and September 2015), random sampling |
| Australia | - 2019: point surveillance study (possibility of being entered all year round, possibility of being selected once a year or repeated several times a year)  - For data collection, although a hospital-wide prevalence survey is recommended, repeated survey is recommended for less than 100 beds and random sampling for more than 100 beds (however, when performing random sampling, at least 50% of inpatients should be included). |

- Methods for selecting assessment subjects that can be implemented at present ( )

- Long-term/ideal goals ( )

- We would like to ask you for any other opinions related to this topic. ( )

**9. We would like to ask for your opinion on the methods for antibiotic quality assessment.**

| Examples |
| --- |
| - Utilisation of standard indicators for assessing the appropriate use of antibiotics (e.g., performing culture tests before using antibiotics, using empirical antibiotics according to guidelines, etc.)  - Relying on expert judgement |

| ROK (policy research service project) | - 2018: expert judgement (investigated some standard indicators)  - 2019: expert judgement, standard indicators  - 2020: utilisation of standard indicators |
| --- | --- |
| United States | - Standard indicators are not specified, but the assessment items are presented as standard indicators.  - Each indicator was presented as supportive/unsupportive and evaluated as a supportive prescription when all indicators were satisfied. |
| Australia | Evaluated utilising standard indicators  - Hospital NAPS: indication documented rate for antibiotic use in medical notes, review for antibiotic use dates in medical notes and documented rate of discontinuation date for antibiotics, prophylactic antibiotic use rate in surgeries for longer than 24 hours, compliance with antibiotic prescription according to therapeutic guidelines, and adequacy ratio of antibiotic prophylaxis  - Long-term care NAPS: indication documented rate for antibiotic use in medical notes and review for antibiotic use dates in medical notes and documented rate of discontinuation date for antibiotic use  - Surgical NAPS: Key quality indicators were not provided. |
| Global PPS project | - In the Global PPS, the person entering the information does not make the final judgement on the appropriate use of antibiotics.  - Instead, an item evaluates whether the antibiotic type is selected in compliance with the guidelines. Additionally, for definitive antibiotics, assessment of drug-bug mismatch is possible as there is an item to enter the antibiotic susceptibility result of the identified bacteria.  - Since information on the route of antibiotic administration and dosage is collected, adequacy assessment is possible for these items during post-hoc analysis. |

- Appropriate methods for assessment at present ( )

- Long-term/ideal goals ( )

- We would like to ask you for any other opinions related to this topic. ( )

**10. In 2019, the Delphi survey with a multidisciplinary expert panel selected the following key indicators for therapeutic, surgical prophylactic, and medical prophylactic antibiotics.**

Table. Results of the selection of key indicators for adequacy assessment of antibiotic prescription in ROK

| **Inpatient**/**emergency room** |
| --- |
| (1) Empirical antibiotic types are prescribed based on (institutional, national, or international) guidelines. |
| (2) If culture results are available, appropriate antibiotics are administered accordingly. |
| (3) A culture test is performed with a sample of the suspected region of infection before or immediately after administering antibiotics for therapeutic purposes. |
| (4) Two or more pairs of blood culture tests are performed before administering antibiotics for therapeutic purposes. |
| (5) The antibiotic dosage or administration interval is adjusted depending on the renal function. |
| (6) The rationale and plan for prescribing antibiotics are registered in the medical record. |
| **Outpatient** |
| (1) Empirical antibiotic types are prescribed based on (institutional, national, or international) guidelines. |
| (2) If culture results are available, appropriate antibiotics are administered accordingly. |
| (3) The antibiotic dosage or administration interval is adjusted depending on the renal function. |
| **Surgical prophylactic antibiotics** |
| (1) Surgical prophylactic antibiotics are prescribed based on (institutional, national, or international) guidelines. |
| (2) Surgical prophylactic antibiotics are administered within 1 hour before surgical incision. |
| (3) Surgical prophylactic antibiotics are discontinued within 1 day after surgery. |

In addition, Australia selects ‘indication documented rate for antibiotic use in medical notes, review for antibiotic use dates in medical notes and documented rate of discontinuation date for antibiotic use, prophylactic antibiotic use rate in surgeries for longer than 24 hours, compliance with antibiotic prescription according to therapeutic guidelines, and adequacy ratio of antibiotic prophylaxis’ as key indicators and reports them annually as follows.


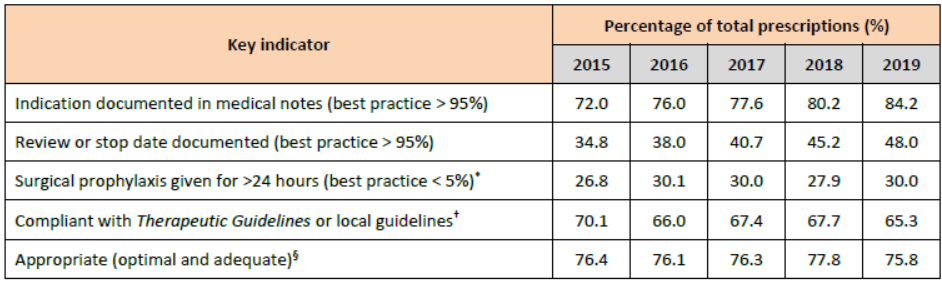


We would like to ask for your opinion on specific measures to utilise the key indicators (Table 2) developed in ROK when regularly evaluating the appropriate use of antibiotics in the future.

( )

- We would like to ask you for any other opinions related to this topic. ( )

**11. We would like to ask for your opinion on methods to encourage organisations to engage in qualitative antibiotic assessment (e.g., reflecting medical quality assessment/accreditation evaluation, paying antibiotic stewardship fee, etc.).**

- Methods applicable at present ( )

- Long-term/ideal goals ( )

- We would like to ask you for any other opinions related to this topic. ( )

**12. We would like to ask for your opinion on reporting/feedback methods for antibiotic quality assessment results.**

| ROK (policy research service project) | - Report of the committee on antimicrobial resistance management of the Korea Disease Control and Prevention Agency (KDCA), conference presentation  - Research paper presentation |
| --- | --- |
| United States | - Introduction to related studies on the CDC website  <https://www.cdc.gov/hai/eip/antibiotic-use.html>  - Research paper presentation |
| Australia | - Hospital NAPS: Participating hospitals can check the results online. For benchmarking, it is possible to compare the results of institutions filtered depending on the region where the medical institution is located, the degree of accessibility of the medical institution, the operator of the medical institution, and the size of the hospital.  - Long-term care NAPS, Surgical NAPS: Although data can be accessed utilising the report results, information on the results of individual institutions or the possibility of benchmarking of similar institutions could not be identified online. |
| Global PPS project | - There are no official results reported. The results are analysed aperiodically and published in medical journals in the form of papers. |

- Methods applicable at present ( )

- Long-term/ideal goals ( )

- We would like to ask you for any other opinions related to this topic. ( )

**13. We would like to ask for your opinion on methods to encourage improvement in antibiotic use by linking antibiotic quality assessment results to an antibiotic stewardship program (ASP).**

| ROK | - There are no specific examples. |
| --- | --- |
| United States | - There are no specific examples of the application of an ASP.  - Repeated surveys in 2011 and 2015 provided motivation: the prevalence of HAIs was investigated to find any changes. As a result of the survey in 2015, it was identified that the prevalence of HAIs decreased from 4.0% to 3.2% compared to the survey in 2011 (P <0.001). The decrease was due to the decreased prevalence of surgical site infections and urinary tract infections. It was identified that the risk of developing HAIs in patients decreased by 16%. |
| Australia | [Hospital NAPS]  - Highlighting priority areas and delivering them to regional hospitals to address the problems found in the qualitative assessment results.  - Encouraging the hospital’s antibiotic stewardship professional group to review results regularly and implement strategies to increase the adequacy of antibiotic use, targeting prescribers and departments with high levels of inappropriate prescription.  - Promoting accessible treatment guidelines for specific infectious diseases and decision-making tools.  - Collaborating with relevant professional groups and academic societies to improve the adequacy of antibiotic use for diseases with low adequacy (e.g., community-acquired pneumonia and chronic obstructive pulmonary disease).  - Developing particular management guidelines for antibiotics with a high rate of inappropriate prescriptions (e.g., amoxicillin-clavulanic acid).  - Developing strategies and guidelines to improve inappropriate prescribing of antibiotics by collaborating with states, territories, and clinical expert groups.  [Long-term care NAPS]  - Periodically reviewing antibiotic selection, duration of use, and use of prophylactic antibiotics in accordance with the guidelines for antibiotic prescription in Australia.  - Sharing the survey results with managers in long-term care facilities, clinicians, pharmacists, and nurses to encourage the institution to develop targeted improvement strategies.  - Using the drugs chart consistently with the National Residential Medication Chart to improve documentation associated with antibiotic prescription.  - Establishing policies that can improve PRN prescriptions (default value, fixed prescription duration, and mandatory review date required).  - Considering hiring external experts for antibiotic prescription and diagnosis, prevention, and management of infections in long-term care facilities.  - Emphasising the necessity for resources and training to implement antibiotic stewardship.  [Surgical NAPS]  - Collaborating with the Royal Australasian College of Surgeons (RACS), surgical professional societies, and other key stakeholders to develop strategies for improving the use of surgical prophylactic antibiotics.  - Providing information to universities, surgical professional societies, states, territories, and private healthcare service providers on the adequacy of prescribing for their specialty.  - Continuously encouraging compliance with the guidelines for antibiotic prescription in Australia.  - Promoting constant monitoring of the adequacy of antibiotic use for surgical prophylaxis in medical institutions in collaboration with states, territories, and private sectors.  - Encouraging continuous monitoring data use to identify targets for improvement and develop and implement improvement programmes. |
| Europe | - There are no specific examples. |
| Global PPS project | - Identifying areas for improvement in relation to antibiotic use utilising assessment and encouraging improvement activities: approximately 97% of participating hospitals found areas that required improvement in antibiotic use by participating in this project, and approximately 69% of participating hospitals performed new antibiotic intervention activities through this. |

- Methods applicable at present ( )

- Long-term/ideal goals ( )

- We would like to ask you for any other opinions related to this topic. ( )

**14. We would like to ask for your opinion on the support required at the national level if periodic qualitative antibiotic assessments are implemented.**

- Required support at present ( )

- Long-term/ideal goals ( )

- We would like to ask you for any other opinions related to this topic. ( )

**15. We would like to ask if any topics not covered in this survey need to be discussed to establish a framework for qualitative antibiotic assessment at the national level.**

(
