# Supplementary material for: Developing a framework for regular and sustainable qualitative assessment of antibiotic use in Korean medical institutions: a Delphi study
Source: Antimicrob Resist Infect Control. 2023 Oct 18;12:114. doi: 10.1186/s13756-023-01319-8 (PMC10585816; doi:10.1186/s13756-023-01319-8)
Supplement: Supplementary file 2 — Additional file 2. Table S1: The response rate of the expertise panel in each round. Table S2: Proposed plan for qualitative assessment of antibiotic use. Table S3: How to best use assessment data. [file 13756_2023_1319_MOESM2_ESM.docx]

**Supplemental Table 1.** The response rate of the expertise panel in each round.

|  | Total | First round | Second round | Panel meeting | Third round |
| --- | --- | --- | --- | --- | --- |
| Experts from the antibiotic resistance committee in the Korean Society of Infectious Diseases | 5 | 5 | 5 | 5 | 5 |
| Experts from the Korea National Antimicrobial Use Analysis System | 4 | 4 | 4 | 4 | 4 |
| Adult infectious disease specialists | 5 | 5 | 5 | 4 | 5 |
| Paediatric infectious diseases specialists | 2 | 2 | 2 | 2 | 2 |
| Experts on policy regarding antibiotic resistance representing the government | 3 | 2 | 2 | 2 | 2 |
| Pharmacist from the Korea Society of Health-System Pharmacists | 1 | 1 | 1 | 1 | 1 |
| Response rate (%) | 20 | 19 (95%) | 19 (95%) | 18 (90%) | 19 (95%) |

**Supplemental Table 2.** Proposed plan for qualitative assessment of antibiotic use.

|  | What is possible at this point | Long-term goals |
| --- | --- | --- |
| Target institutions | Tertiary, Secondary, Primary-care hospitals | Tertiary, Secondary, Primary-care hospitals  Long-term care hospitals  Specialty hospitals (orthopaedics, ophthalmology, etc) |
| Interval | Difficult to find consensus | At least once a year |
| Type (Systemic, Local) | Systemic antibiotics (IV, oral) | |
| The range of antimicrobial agents that can be included | Antibacterial agents | Antibacterial agents  Antifungal agents |
| Administration route | IV, oral route | |
| Types of antibiotics based on the purpose of the antibiotic prescription | Therapeutic, surgical, and medical prophylaxis | |
| Where the infection was acquired | Antibiotics for community acquired and healthcare-associated | |
| Evaluation methods | Evaluation using systematised criteria (standard indicators) | |
| Extraction methods | Randomised evaluation of antibiotics prescribed in a specific time period, with evaluation of specific antibiotics or conditions as needed.  Randomised evaluation of antibiotics prescribed on a specific day  Evaluation limited to specific conditions or specific antibiotics | Evaluation of antibiotics prescribed in a specific time period, either randomly or by census, with evaluation of specific antibiotics or conditions as needed. |

**Supplemental Table 3.** How to best use assessment data.

|  | How to report/feedback | How to connect with antibiotic stewardship programs |
| --- | --- | --- |
| Agreed | 1) Disclosure of qualitative assessment results along with quantitative assessments, antibiotic resistance rates, etc. through publicly available antibiotic use/resistance reports (anonymised without disclosing institutional names).  2) Individual medical institutions can view institutional, regional, and national results through a private website (anonymised without disclosing institutional names).  3) Disclose regional and national results by adding them as indicators to publicly available sites such as the Health Insurance Review and Assessment Service's website (individual institutional results are not disclosed). | 1) Reporting of quality assessment results by individual healthcare providers to management/staff within the healthcare organisation.  2) Policy and institutional approaches to address common antibiotic misuse identified in the quality assessment (e.g., incentives, inclusion in quality measures, etc.)  3) Implementation of healthcare provider education (e.g., mandatory medical association education) for common antibiotic misuse identified in the qualitative assessment.  4) Publicising and disseminating the results of the antibiotic quality assessment analysis to the public. |
| Not agreed | 1) Publish an annual report or publicly available website that discloses individual organisations' inappropriate prescribing rates to the public  2) Maintaining a website where individual healthcare organisations can view only their own results. | None |
